# Fast batch searching for protein homology based on compression and clustering

**DOI:** 10.1186/s12859-017-1938-8

**Published:** 2017-11-21

**Authors:** Hongwei Ge, Liang Sun, Jinghong Yu

**Affiliations:** College of Computer Science and Technology, Dalian University of Technology, No.2, Linggong Road, Dalian, China

**Keywords:** Protein homology, Batch searching, Compression, Clustering

## Abstract

**Background:**

In bioinformatics community, many tasks associate with matching a set of protein query sequences in large sequence datasets. To conduct multiple queries in the database, a common used method is to run BLAST on each original querey or on the concatenated queries. It is inefficient since it doesn’t exploit the common subsequences shared by queries.

**Results:**

We propose a compression and cluster based BLASTP (C2-BLASTP) algorithm to further exploit the joint information among the query sequences and the database. Firstly, the queries and database are compressed in turn by procedures of redundancy analysis, redundancy removal and distinction record. Secondly, the database is clustered according to Hamming distance among the subsequences. To improve the sensitivity and selectivity of sequence alignments, ten groups of reduced amino acid alphabets are used. Following this, the hits finding operator is implemented on the clustered database. Furthermore, an execution database is constructed based on the found potential hits, with the objective of mitigating the effect of increasing scale of the sequence database. Finally, the homology search is performed in the execution database. Experiments on NCBI NR database demonstrate the effectiveness of the proposed C2-BLASTP for batch searching of homology in sequence database. The results are evaluated in terms of homology accuracy, search speed and memory usage.

**Conclusions:**

It can be seen that the C2-BLASTP achieves competitive results as compared with some state-of-the-art methods.

## Background

The task of batch searching for protein homology often arise in the field of bioinformatics. As the exponential growth [1, 2] of protein databases, searching for homologs often become ineffective due to the intensive computational efforts involved [3]. For example, in order to investigate the homology of a new protein sequence set, a cross-species protein identification method needs to search millions of sequences in the NR database. Moreover, since the public databases (such as PDB [4], NR [5], and SWISSPORT [6]) are continuously updated, the task of homology search is becoming more computationally expensive and redundant. With the increasingly number of the users and queries being accessible to the public databases, the query tasks are becoming heavy and heavy. Thus effective algorithms that match sets of protein query sequences in large-scale sequence datasets are always in demand.

BLAST [7] will take a longer time when the scale of query set is getting larger since it evaluates a single query once. It alternatively employs a brute force approach to compare query sequence and database sequence. More specially, the BLAST searches for short fixed-length word pairs in the sequences and then extends them to higher-scoring regions. For each query sequence, the algorithm scans the entire database and compare database sequence with the querying one to find the subsequences. The BLAST maybe conduct reduplicative scans to find common subsequences. Thus, there is an urgent need for a tool that can significantly speed up batch homology searching.

There are many efforts that develop relative techniques for efficient homology searching. MegaBLAST [8] is a greedy sequence alignment algorithm. It is faster than basic BLAST, but it is less effective for aligning highly similar sequences with larger size. MPBLAST [9] concatenates queries by grouping them into a single query, with the objective of reducing times of database accessing. BLAST++ [10] transforms a collection of queries into a single virtual query, which guarantees the seed searching process to be performed once for common subsequences. However, it does not take the redundancy of database into consideration, and will get inefficiency when applied in large-scale database. The BLAST+ [11] is developed based on the advanced results from MPBLAST, BLAST++, miBLAST [12], BLAT [13]. However, its performance is unsatisfactory for batch queries when applied to search on large-scale dataset. MpiBLAST [14] speeds up homology search by using parallel processing technique on a cluster of machines. CUDA-BLASTP [15] utilize GPU to speed up searching, however, it is not suitable for supporting large-scale databases due to the limit of memory size. Following the mechanism of CUDA-BLASTP, several homology search tools have been developed, such as RAPSearch [16] and GHOSTZ [17]. However, these methods require more space to retain relative information of sequences, which incurs excessive memory and storage cost. So, the problem of batch searching for protein homology still remains challenging and there remains much room for researchers to improve their algorithms.

In this paper, we conduct studies with the objective of improving the performance of batch homology search, and a fast compression and clustering based BLASTP (C2-BLASTP) algorithm for large-scale protein homology search is proposed. Firstly, the query set and the database are compressed to reduce sequence redundancy. Then a new database is clustered according to the Hamming distance of similar subsequences. The objective is to minimize the computation time on ungapped extensions. Furthermore, an execution database is constructed, on which the homology search is performed. The execution database is considered as a collection of all the potential homologous sequences.

## Methods

An effective strategy to improve the efficiency of batch query is to reduce the redundant sequences in query set and the database. The underlying mechanism works by finding representative sequences to express the information throughout the sequence sets. To guarantee the search precision and speed, the representative sequences are expected to be non-redundant as well as to express complete information. The proposed fast batch homology search algorithm (C2-BLASTP) has three major components, i.e., the compression, the clustering, and the batch searching. In the compression process, the database and the query set are compressed by removing the subsequences with high similarity, and leaving the representative subsequences remained. In the clustering process, the subsequences in the compressed database is further grouped based on their similarities, and the potential hits will be obtained. In the batch searching process, a small scale executable database is constructed by the potential homology hits, and the homology search is performed in the execution database. The details above three components are presented in the following subsections.

### Compression

In the phase of compressing, the associations among potential highly similar subsequences are setup by a mapping between seeds and subsequences, where seed refers to a segment of protein sequence with five amino acids, and subsequence refers to a fraction of protein sequence. The similarity among the subsequences that point to the same seed is evaluated by Needleman-Wunsch [18]. The highly similar subsequences are grouped into one cluster, with one appropriate subsequence being retained as its representation. By applying this mechanism, the data redundancies can be reduced. Meanwhile, the query sequence and database can be compressed.

More specifically, the compression process for query set and protein database is executed as follows.

**Fig. 1:**
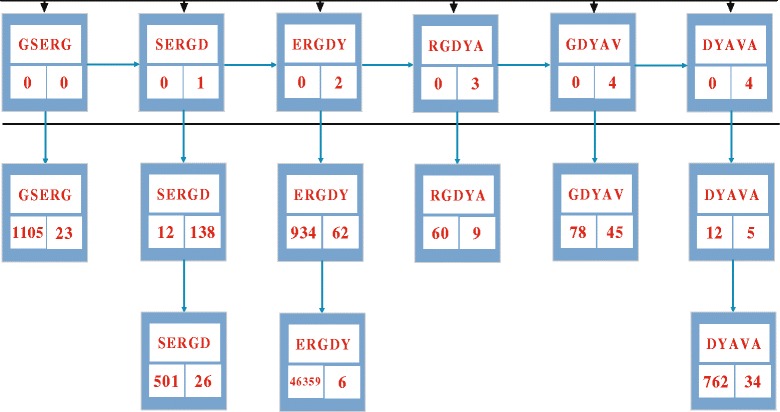
Structure of key-entry pair map. This is an example of the key-entry pair map structure. Each key in the map is a segment of protein sequence with five amino acids, and it is also called a seed. Each entry has three attributes, i.e., sequence number, starting amino acid position, and the link to the next sequence. The algorithm scans the first protein sequence from left to right and groups every five amino acids into a key

1. An initial key-entry pair map structure is constructed. Each key in the map is a segment of protein sequence with five amino acids, and it is also called a seed. The attributes of the key include an index number in the database (also referred as sequence number), a starting amino acid position, and a link to the next subsequence. By scanning the protein sequence from left to right, a key is created using every five amino acids. Figure 1 shows an example of the key entry pair map structure.
2. Each sequence in the query set or the protein database is compared with the existing keys in the current map. By scanning the input protein sequence from left to right, the keys are compared with every five successive amino acids. If the compared segment matches one of the existing keys, the Needle man Wunsch algorithm is carried out, the segment will be truncated starting from the current position, and will be connected with other segments that are linked by the matched key. Otherwise, a new key will be added, and its corresponding entry attributes will be added to the current map.
3. Redundant segments in sequences are compressed. Similarity can be computed according to the alignment result using BLOSUM62 [19]. When the similarity is higher than a given threshold (80%), the referred subsequence is considered to be redundant. So the subsequence is deleted, meanwhile, a new link to the current key is added and the difference between the two subsequences is recorded in a special script.
4. A final non-redundant segment pool is created. The new database consists of non-redundant segments of protein sequence and the corresponding sequence information.

**Fig. 2:**
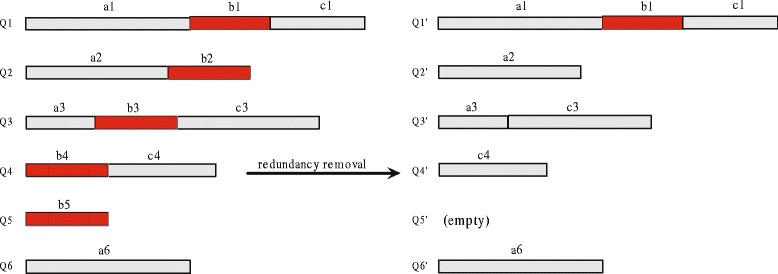
An example for redundancy removal. This is an example for redundancy removal. Q1 to Q6 are six sequences in query set or database. The red shadow segments are subsequences with more than 80% similarity. By conducting redundancy removal, Q2’ is generated by deleting similar segment b2 in the rear of Q2; Q3’ is generated by concatenating a3 and c3 as well as deleting similar segment b3; Q4’ is generated by deleting similar segment b4 in the front of Q4; Q5 is completely removed; Q6 is completely reserved

The above compression process includes redundancy analysis, redundancy removal and distinction record. The redundancy analysis is implemented using the key-entry pair map and the alignments. Figure 2 presents an example of redundancy removal. Q1 to Q6 are six sequences. The red shadow segments are subsequences with more than 80% similarity. By conducting redundancy removal, Q2’ is obtained by deleting similar segment b2; Q3’ is obtained by concatenating a3 and c3 as well as deleting similar segment b3; Q4’ is obtained by deleting similar segment b4; Q5 is completely removed; Q6 is completely reserved.

**Fig. 3:**
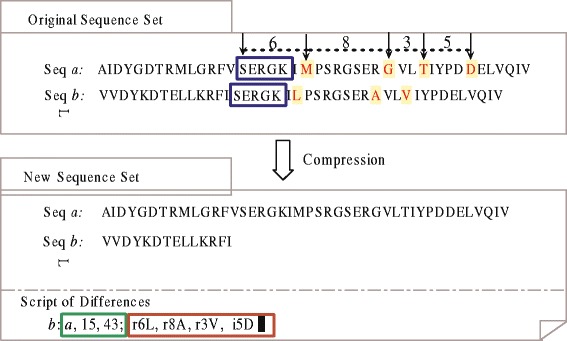
Illustration of compression process. This is an illustration of compression process. Seq *a* and seq *b* are sequences taken from the original sequence set which include the same key ‘SERGK’ with their subsequences similarity being more than 80%. Seq *b* is compressed by removing the similar counterparts. To keep the completeness of seq *b*, a script is employed to record the differences between seq *a* and the compressed seq *b*, where ‘*a*, 15, 43’ records the site of the removed segment, ‘r6L, r8A, r3V, i5D’ records the small differences compared with the representative sequence

To keep the completeness of the sequence information, the small differences (less than 20%) among the similar subsequences are recorded using a script. Figure 3 presents an illustrative example of compression. Seq *a* and seq *b* are sequences taken from the original sequence set which include the same key ’SERGK’. After the key, the similarity of their two subsequences is more than 80%. So seq *b* is compressed by removing the similar counterparts. To avoid losing pseudo redundancy in the remaining segment, a script is employed to record the small differences. The contents of the record include pairs of position information and distinction information. For example, a section of ‘*a*, 15, 43’ indicates the representative sequence is seq *a*, and the compressed segment starts at the 15th residues and ends at the 43rd residues. A section of ‘r6L, r8A, r3V, i5D’ indicates the small differences compared with the representative sequence. The lower-case letters *r*, *i*, and *d* denote the three operations of replacement, insertion and deletion, respectively. The digit either denotes the distance between the current mismatching residue and its nearest mismatching predecessor, or the distance between the first mismatching residue and the initial position of the key. The capital letter denotes the actual residue in the compressed redundant subsequence. Thereafter, the original sequence can be recovered using the information in the difference script. Besides, the compressed sequence database is written in FASTA format. Algorithm 1 gives the pseudo-code of compression.

### Clustering

By conducting the compression process, the redundancy in the query set and the protein database can be reduced. However, since the compressed protein database is still large as the fast growing of protein sequences, the online running of BLASTP is still time consuming. Moreover, the traditional BLASTP takes much time extending alignments without gaps because of the large number of seeds (including 3 amino acids). The C2-BLASTP further conduct clustering on the compressed database. Following this, the process of hits finding is implemented on the representative seed of each cluster.



**Fig. 4:**
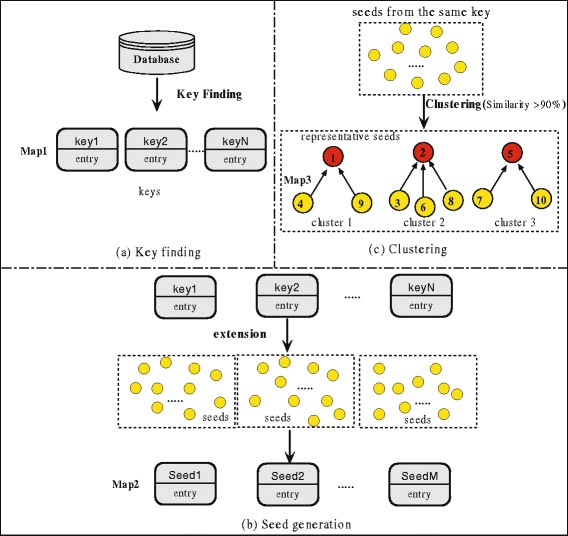
Generation process of clustered database. This figure shows the clustering process. In the key finding process, the key-entry map is created by conducting compress operation on the database. The length of the key is automatically selected based on the BLOSUM62 matrix. In the seed generation process, the seeds are generated by extending from the keys and the seed-entry map is created. And in the clustering process, a representative seed is selected for each cluster, to which other seeds are linked, and the clustering map is created

To further improve the sensitivity and selectivity of pairwise sequence alignments, ten groups of reduced amino acid alphabets (A, {K, R }, {E, D, N, Q }, C, G, H, {I, L, V, M }, {F, Y, W }, P, {S,T }) that are statistically derived based on the BLOSUM62 matrix are used. In essence, the similar amino acids are implicitly grouped together. The clustered database is obtained by the processes of key finding, seed generation, and clustering, which is illustrated in Fig. 4.

How to determine the key length is crucial in key finding task. In fact, the short subsequences of the same length tend to appear with different frequencies in the database because of the composition bias in biology. It has been validated that the keys with 6-9 amino acids tend to appear with higher efficiency [16]. So, the lengths of keys are automatically selected in the range of 6-9 amino acids based on the sum of the match scores of the short subsequences. The match score is obtained by the BLOSUM62 score matrix and is taken by the highest score in each group of amino acids. To avoid insignificant short segments, the threshold *T* is taken empirically with value 39. When the sum of match scores for short subsequences exceeds *T*, the subsequence is considered as a key. For example, the subsequence ‘YKWVN’ is not used as a key because its score sum is less than 39, while ‘YKWVNK’ is used as a key because its score higher than 39. If a key is obtained, then a key-entry map is created and extended by following a similar procedure in compression process. Finally, a complete key-entry map (Map1) for all of the keys can be obtained.

Next, seeds can be generated from keys. The seeds are composed of ten residues, with the first five residues being extended forward from the starting point of the key, and the remaining residues being taken from the first five residues of the key. Finally, the seeds produced from the same key are clustered according to Hamming distance, respectively. The seeds will be group into one cluster if their similarity exceeds a given threshold (90*%*). Each cluster has one representative seed, with other seeds being linked to. Meanwhile, two association diagrams are created. The first diagram is the seed-entry map for the representative seed (Map2), and its entry includes the cluster ID and the location of representative seed. The other diagram is the clustering map (Map3). As shown in Fig. 4 c, the diagram describes the cluster ID and the location of its cluster member. The above procedure accelerates the search speed since it groups similar subsequences together.

### Batch searching

The clustered database is constructed offline by implementing the operators of compression and clustering. It needs to be updated regularly as the database expanses. For given query sequences, the objectives lie with finding enough information for homology from the clustered database, and creating a smaller scale execution database. The execution database is a collection of all the potential homologous sequences with which the homology search can be performed.

Since hits associate with potential homologous sequences, how to find hits from the clustered database plays an important role in constructing the execution database. Hits are the set of results obtained by searching the clustered database using compressed query set as index. To compare query sequences with the clustered database that is described by three maps in “Clustering”section, we construct the seed-entry map for query set and keep their format being consistent. More specifically, the query sequences are firstly re-expressed by the reduced amino acid alphabets, and then every ten adjacent residues are taken as a seed in the query set directly. Thereafter, we compare each seed in query set with the representative seeds in Map2. If they are identical, the corresponding original fragments (non-reduced amino acid alphabets) can be recovered according to their entries in maps. So, the similarity between the fragment of query sequences and the cluster representative can be calculated. If the similarity exceeds a given threshold (80*%*), all the members in the cluster can be obtained by the cluster ID. Then we conduct gapped and ungapped extensions to obtain hits.

When the similarity is less than the threshold, the query seed may still be of highly similar with other elements of the cluster due to the existing differences between the cluster representative and its members. In this case, the compensation analysis is further conducted by employing triangle inequality [17], so that the search accuracy can be improved. The formulation is as follows.

1 $$\begin{array}{*{20}l} d(S_{q},S_{m})\geq{d}(S_{q},S_{r})-d(S_{r},S_{m}) \end{array} $$

Where *S*_*q*_, *S*_*m*_ and *S*_*r*_ are the query seed, the cluster member, and the cluster representative, respectively. *d*(*S*_1_,*S*_2_) is the distance between seed *S*_1_ and seed *S*_2_. In particular, the maximum value of *d*(*S*_*r*_,*S*_*m*_) is 1 because the cluster threshold *T*_*c*_ is taken as 90%. So, the lower bound of distance between *S*_*q*_ and *S*_*m*_ can be obtained. If the lower bound is less than or equal to the distance calculated from similarity threshold *T*_*s*_, then the query seed may be highly similar to the member seed. Therefore, we conduct gapped and ungapped extension to get hits.

**Fig. 5:**
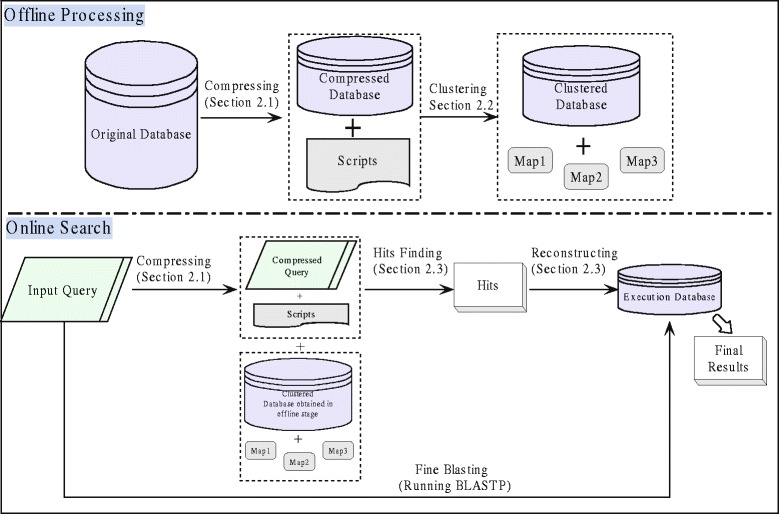
The framework of C2-BLASTP. This figure shows the framework of the C2-BLASTP. In the offline processing step, the original database is compressed, and further grouped into clusters. In the online searching step, the input query set is compressed, then the hits set is obtained by running BLASTP on the compressed query set and the compressed database. Following this, the hits related redundancy sequences are assembled to form an execution database. Finally, batch searching is conducted between the original query set and the execution database using BLASTP

The hit set is composed of non-redundancy subsequences in the compressed database. Further, by utilizing the scripts of the compressed database, all the key related redundancy sequences from the original dataset can be assembled to form a final execution database. Finally, batch searching for protein homology can be conducted between the original query set and the execution database using BLASTP. In summary, the framework of the proposed C2-BLASTP algorithm is shown in Fig. 5.

## Results and discussion

### Experimental datasets and settings

In this section, experiments are conducted to evaluate the performance of the proposed C2-BLASTP. In the experiments, the NR database built on June 2013 is taken as benchmarks. The database has 26.7 million protein sequences, including a total of 9.3 billion amino acids. We randomly select a certain number of sequences from the Saccharomyces Genome Database (SGD) and the ENV _NR Database as query sequences. The SGD contains the proteomes of 21 strains of yeast [20]. The ENV _NR contains some translations from the ENV.NT (nucleotide) database, and the ENV.NT contains DNA sequences from the environment directly. The organization of the datasets indicates the varieties of their organisms. The proteins from environmental projects are presented in either the NR or the ENV _NR database, depending upon whether that sequence has been identified as a particular organism (NR), or the organism is unknown (ENV _NR). All the experiments are carried out on a work station with dual 4-core Intel Xeon E-2609 processor, 32 GB memory and using Centos Linux.

### Existing algorithms for comparison

For the purpose of comparison, we select the following classical or state-of-the-art batch searching algorithms.

1. BLASTP (BLAST+ version 2.2.31): BLASTP (Basic Local Alignment Search Tool for Protein) can be used to infer functional and evolutionary relationships among sequences. The executing process include word matching, ungapped extension, and gapped extension. The algorithm can be used to compare protein sequences with sequence databases and to calculate the statistical significance of matches, and it also can be used to infer functional and evolutionary relationships among sequences.
2. CaBLASTP [21] (Version 1.0.3): CaBLASTP introduces compression strategy and achieves a faster speed than BLAST by searching in the compressed database. It firstly searches the protein homology in a coarse database where the redundant subsequences are removed, and then uses the obtained initial results to search the original database for similar sequences.
3. GHOSTZ [17] (Version 1.0.0): GHOSTZ uses the strategy of clustering database subsequence and filters out the non-representative seeds within these clusters to minimize the computation time spent on ungapped extensions.

### Effects of compression

In this section, to test the compression performance of the C2-BLASTP, we conduct experiments on the NR database and the Saccharomyces Genome Database. The compression threshold *T*_*t*_ is an important parameter in the process of compressing redundant segments in query set. In the experiment, we set the threshold *T*_*t*_ empirically. The algorithm is executed repeatedly, with *T*_*t*_ value taken as 40%, 60%, 80% and 100%, respectively. On the other hand, the compression threshold for the segments in the retrieved NR database is empirically taken as 80%. The query set is composed of 100 randomly selected protein sequences from SGD, and the searching for protein homology in the NR database is conducted by using C2-BLASTP. The algorithm is repeated 10 times independently and the average results are presented in Table 1. In Table 1, the number of the amino acids after compressing, the running time (s), true positive rate (TPR), false positive rate (FPR), the acceleration ratio (AR) and the compression ratio (CR) are presented. The TPR reflects the hits found by both the C2-BLASTP and the BLASTP. The FPR reflects the hits found by C2-BLASTP but not found by BLASTP. Because we search for the protein homology between the original query set and the execution database using BLASTP, the false positives with respect to the original BLASTP are zero. From Table 1, it can be seen that the number of amino acids in the uncompressed query set is 53978, whereas the number of their compressed counterparts is 38549, 36508 and 31572 by taking the compression thresholds as 80%, 60% and 40%, respectively. And the corresponding compression ratio is 0.71, 0.68 and 0.59, respectively. The number of the amino acids in the original NR database is 9.4 billion, whereas their counterpart is 3.6 billion in the compressed database, which is only 38% of the original scale. The high compression ratio for the NR database is caused by the local similarity, even though there is no high redundancy of the global sequence-identity. So, the computation time can be reduced. It can be seen that the acceleration ratio is 12.6 when only the NR database is compressed. Moreover, the acceleration ratio reaches 13.1, 14.1 and 16.6 when the query set is compressed with different threshold *T*_*t*_. Meanwhile, we can achieve high TPR values with respect to BLASTP.

**Table 1:** Comparison results using different compression threshold for the C2-BLASTP

*T*_*t*_ for query set	amino _acids	time (s)	TPR (%)	FPR (%)	AR	CR
C2-BLASTP (40%)	31572	180.9	94.3	0	16.6	0.59
C2-BLASTP (60%)	36508	212.9	96.3	0	14.1	0.68
C2-BLASTP (80%)	38549	229.2	96.5	0	13.1	0.71
C2-BLASTP (100%)	53978	238.2	97.6	0	12.6	1.0
BLASTP	53978	2994.4	100	0	1.0	1.0

### Comparison with other methods and analysis

In this subsection, the results of the C2-BLASTP on the NR database is presented. Single sequence, 30 sequences, 100 sequences, 200 sequences, 500 sequences and 1000 sequences that are randomly chosen from the ENV _NR are taken as the query set. The results are compared with BLASTP, CaBLASTP and GHOSTZ, respectively. For each query, the experiment is repeated 10 times, and the results are presented in Table 2.

**Table 2:** Comparative results of the C2-BLASTP with other algorithms

Query Seq	BLASTP	CaBLASTP			GHOSTZ			C2-BLASTP		
	Time (s)	Correct (%)	Alignment	Time (s)	Correct (%)	Alignment	Time (s)	Correct (%)	Alignment	Time (s)
			accuracy (%)			accuracy (%)			accuracy (%)	
Single	300.9	100	100	219.7	100	100	448.2	100	100	78.1
30	448.3	100	100	472.8	100	96.7	464.2	100	100	89.3
100	2271.9	100	100	1373.1	98.0	93.9	492.9	99.0	100	149.7
200	4167.4	100	100	2292.5	96.5	95.3	514.6	98.0	100	271.6
500	9028.2	99.2	100	5674.9	83.6	86.4	593.5	95.1	100	1551.4
1000	18016.2	99.0	100	11340.5	77.8	84.6	915.3	94.6	100	2562.1

**Fig. 6:**
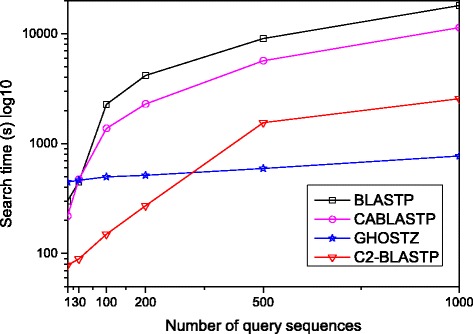
Runtime curves obtained by different algorithms. This figure presents the average runtime curves of the C2-BLASTP and the compared algorithms

The runtime listed in Table 2 refers to the online time for homology search. So, the runtime for BLASTP includes the time spent in the process of seed search and alignment. The runtime for the GHOSTZ includes the time spent in the process of map creation and alignment. The runtime for CaBLASTP includes the time spent in the phases of coarse search, database reconstruction and fine search. Whereas the runtime for C2-BLASTP includes the time spent in the phases of hit finding, database reconstruction and fine search. From Table 2, it can be seen that GHOSTZ and C2-BLASTP are faster than the BLASTP and the CaBLASTP. Moreover, the C2-BLASTP is faster than GHOSTZ when the scale of query set is smaller than 200 sequences. Figure 6 presents the average runtime curves of the C2-BLASTP and the compared algorithms. It can be seen that the search time increases as number of query sequences increases for all the C2-BLASTP and the compared algorithms, and the C2-BLASTP takes the shortest search time when the number of query sequences approximates 300.

The advantage of the GHOSTZ lies in performing seed search in the offline process of database construction. And the representative seeds further improve the search speed. However, the GHOSTZ adopts the reduced amino acid alphabets in the original database, so the more underlying matched seeds will result in the larger number of alignments. When the query set is relatively small, the number of seeds in BLASTP is not so large. In this case, GHOSTZ does not have advantage over other algorithms in terms of speed. Besides, GHOSTZ need more memory requirements during the process of creating clustered database. The C2-BLASTP compress the original database offline at one time, and further the representative seeds are obtained by clustering. Due to such advantages, it outperforms other algorithm with the small-scale query set (<200 sequences) in terms of speed. With the increase of the query sequences, C2-BLASTP spends much time in reconstructing execution database.

**Fig. 7:**
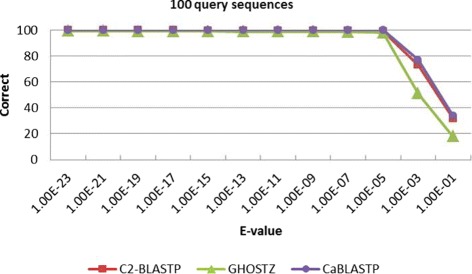
Search accuracy of different methods for 100 query sequences against the NR database

**Fig. 8:**
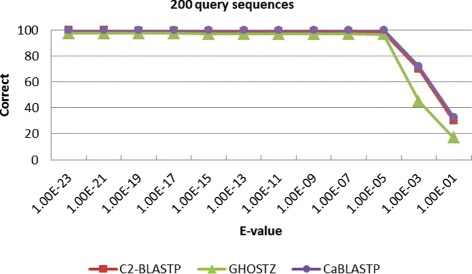
Search accuracy of different methods for 200 query sequences against the NR database

**Fig. 9:**
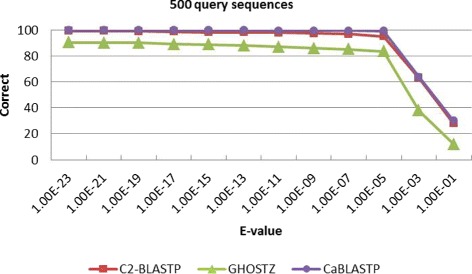
Search accuracy of different methods for 500 query sequences against the NR database

**Fig. 10:**
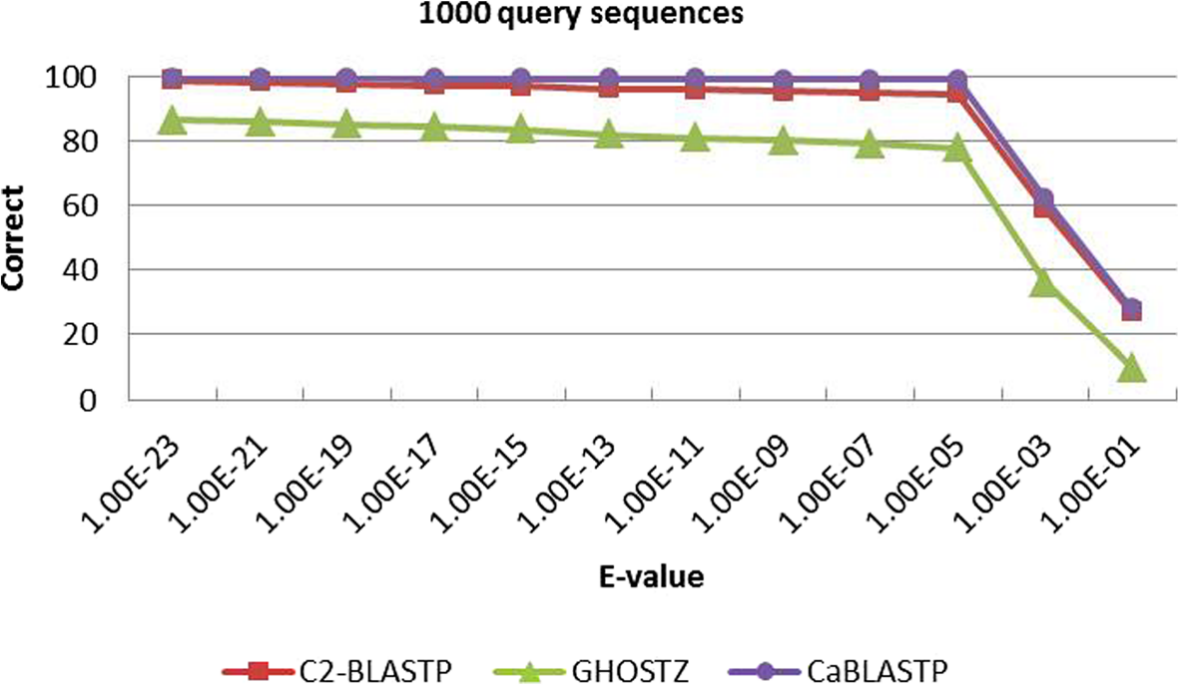
Search accuracy of different methods for 1000 query sequences against the NR database

Meanwhile, to find out the overlap elements, we compare the homology sequences found by C2-BLASTP with those identified by other algorithms. Table 2 lists the correct rate and alignment accuracy of the homology search results obtained by different algorithms. The correct rate reflects the proportion of identical sequences with the highest score that obtained by BLASTP and other algorithms. The alignment accuracy reflects the number of correctly aligned positions that are obtained by both the compared algorithms and the standard BLASTP. From Table 2, it can be seen that the correct overlap of sequence hits is more than 94% and the alignments is 100% by using our C2-BLASTP. In other words, when a hit is found, the alignment perfectly matches the standard BLASTP alignment. To better investigate the impact of E-value on accuracy, more tests about a series of comparison with different E-value thresholds are carried out. We perform batch searching of homology on the NR database, and 100, 200, 500 and 1000 sequences are randomly chosen from the ENV NR as the query set. The results are presented in Figs. 7, 8, 9, and 10. From the tables, it can be seen that when the E-value is below 1.0*E*−5, the C2BLASTP obtains almost the same results with CaBLASTP, and obtains better results than GHOSTZ. In particular, the results are significant better than those of GHOSTZ when the number of query 500 and 1000.

### Analysis of memory and disk cost

With the exponential growth of protein sequence databases, the storage performance becomes an important factor when designing the protein homology search algorithms. The processing capacity of most of personal computers is difficult to keep up with the growing speed. So, some homology search tools provide the corresponding processed sequence database for users, such as CaBLASTP. When the original database is updated, users can add new sequences to the downloaded database by means of a provided function. So, for PC users, the PC memory needs to satisfy the requirements of constructing the database. Besides, the storage capacity of hard disk should be enough to handle the volume of database and the related information. In the proposed C2-BLASTP, the memory requirements mainly incurred in the process of compression and clustering. Due to the reduction of the local redundancy in compression process, C2-BLASTP reduces working memory and disk requirements. GHOSTZ needs more space to retain relative information of sequences based on the original database, while the clustering process of C2-BLASTP only needs to retain the useful information of the non-redundancy database. Therefore, using this technique, C2-BLASTP requires less memory than GHOSTZ (512 M per chunk). When we use the ENV _NR(1.9 GB) as the appended database (query sequence database), as shown in Table 3, C2-BLASTP requires 12.1GB memory and 5.3 GB disk for constructing the database. In contrast, CaBLASTP requires 12.4 GB memory and 2.7 GB disk and GHOSTZ requires 25.3 GB and 9.5 GB disk.

**Table 3:** Costs of memory and disk for appended sequence set using different algorithms

Algorithm	Memory size (GB)	Disk size (GB)
C2-BLASTP	12.1	5.3
CaBLASTP	12.4	2.7
GHOSTZ (512MB)	25.3	9.5

## Conclusions

The rapid growth of the protein sequences in databases makes batch homology search challenging. The proposed C2-BLASTP fully exploits the joint information among the query sequences and the database. To recude redundancies, the queries set and database are compressed based on the Needleman-Wunsch algorithm. And the database is further clustered to reduce the computation time incurred by ungapped extensions. Finally, the homology search is conducted in a constructed execution database, which is considered as a collection of all the potential homologous sequences. In conclusion, C2-BLASTP can be implemented as an extension of the NCBI BLAST+, and can be easily interfaced with other programs that use protein BLAST as search tools. Numerical Experiments on NCBI NR database show that the C2-BLASTP for batch searching of homology in sequence database is effective. C2-BLASTP can also be integrated with different BLAST tools to improve the search speed by replacing BLASTP in the fine BLASTP process.

The future perspectives of this paper are twofold.

1. The current C2-BLASTP will be extended by adapting with high performance hardware such as GPU to accelerate searching speed.
2. As the current C2-BLASTP only realized the high speed batch searching of protein sequence, it will be extended to a wider varieties of toolbox similar to BLAST.

## References

[1] Kahn SD (2011). On the future of genomic data. Science.

[2] Daniels NM, Gallant A, Peng J, Cowen LJ, Baym M, Berger B (2013). Compressive genomics for protein databases. Bioinformatics.

[3] Zepeda G, Reyna C, Fu Y, Rodriguez L, Isabel C (2017). Novel protein interactions with an actin homolog (mreb) of helicobacter pylori determined by bacterial two hybrid system. Microbiol Res.

[4] Nat Struct Biol. 2003; 10:980. doi: 10.1038/nsb1203-980. 10.1038/nsb1203-980 14634627

[5] Pruitt KD, Tatusova T, Maglott DR (2005). Ncbi reference sequences (refseq): a curated non-redundant sequence database of genomes, transcripts and proteins. Nucleic Acids Res.

[6] The UniProt Consortium (2017). Uniprot: the universal protein knowledgebase. Nucleic Acids Res.

[7] Altschul SF, Madden TL, Schaffer AA, Zhang J, Zhang Z, Miller W, Lipman DJ (1997). Gapped blast and psi-blast: a new generation of protein database search programs. Nucleic Acids Res.

[8] Morgulis A, Coulouris G, Raytselis Y (2008). Database indexing for production megablast searches. Bioinformatics.

[9] Korf I, Gish W (2000). Mpblast: improved blast performance with multiplexed queries. Bioinformatics.

[10] Wang H, Oi BC, Tan KL (2003). Blast++: Blasting queries in batches. Bioinformatics.

[11] Camacho C, Coulouris G, Avagyan V (2009). Blast+: architecture and applications. BMC Bioinformatics.

[12] Kim YJ, Boyd A, Tthey BD (2005). miblast: scalable evaluation of a batch of nucleotide sequence queries with blast. Nucleic Acids Res.

[13] Kent WJ (2002). Blat-the blast-like alignment tool. Genome Res.

[14] Darling A, Carey L. The design, implementation, and evaluation of mpiblast. In: The 4th International Conference on Linux Clusters, San Jose. San Jose: 2003. p. 656–64.

[15] Liu W, Schmidt B, Muller-Wittig W (2011). Cuda-blastp: accelerating blastp on cuda-enabled graphics hardware. EEE/ACM Trans Comput Biol Bioinforma.

[16] Ye Y, Choi JH, Tang H (2011). Rapsearch: a fast protein similarity search tool for short reads. BMC Bioinformatics.

[17] Suzuki S, Kakuta M, Ishida T, Akiyama Y (2015). Faster sequence homology searches by clustering subsequences. Bioinformatics.

[18] Needleman SB, Wunsch CD (1970). A general method applicable to the search for similarities in the amino acid sequence of two proteins. J Mol Biol.

[19] Henikoff S, Henikoff JG (2000). Amino acid substitution matrices. Adv Protein Chem.

[20] Cherry JM, Hong EL, Amundsen C (2011). Saccharomyces genome database: the genomics resource of budding yeast. Nucleic Acids Res.

[21] Daniels NM, Gallant A, Peng J, Cowen LJ, Baym M, Berger B (2013). Compressive genomics for protein databases. Bioinformatics.

